# Targeting Innate Immune Cells for Immunotherapy

**DOI:** 10.1155/2017/4271384

**Published:** 2017-04-05

**Authors:** Leandro J. Carreño, Rafael Prados-Rosales, Mercedes López, Andrés Baena, Pablo A. González

**Affiliations:** ^1^Millennium Institute on Immunology and Immunotherapy, Santiago, Chile; ^2^Programa de Inmunología, Instituto de Ciencias Biomédicas, Facultad de Medicina, Universidad de Chile, Santiago, Chile; ^3^Centro de Investigaciones Cooperativas en Biociencias (CIC bioGUNE), Bilbao, Spain; ^4^Departamento de Microbiología y Parasitología, Facultad de Medicina, Universidad de Antioquia, Medellín, Colombia; ^5^Departamento de Genética Molecular y Microbiología, Facultad de Ciencias Biológicas, Pontificia Universidad Católica de Chile, Santiago, Chile

One of the most effective ways to modulate either positively or negatively host immune responses (i.e., to treat cancer and autoimmunity, resp.) is to target effector immune cells, such as B cells, CD4^+^, and CD8^+^ T cells, as well as professional antigen-presenting cells, such as dendritic cells (DCs), that promote the activation of the latter two. Frequently, this modulation is aimed at targeting the initiation of the T cell immune response, a process known as T cell priming, which affects not only T cell outcome but also B cell responses that are influenced by the help of CD4^+^ T cells. At present, several strategies include the optimization of adjuvants and drugs to manipulate the phenotype and function of DCs, frequently with their ex vivo drug treatment and their reintroduction into the host, activating/inhibiting ligands that interact with T cell activation, differentiation, and function. Another strategy consists in ex vivo T cell expansion and reinjection into the host, among others. Although these immunomodulating strategies have shown relatively high success in the clinic with promising therapeutic potential, there are many other strategies that target different immune cell types, especially those associated with the innate immune system, that play crucial roles in immunomodulation. Moreover, it has been evidenced during recent years that cells of the innate immune system can modulate and dictate the inflammatory environment that will take place during T cell priming. Cells of the innate immune system are key players not only at initiating and regulating adaptive immune responses, such as those elicited against pathogens and cancer, but also at modulating tolerance to autoantigens to prevent autoimmune diseases. While some of these cells are considered exclusively innate, as natural killer (NK) cells and innate lymphoid cells (ILCs), others are positioned at the interface of innate and adaptive immune responses, such as DCs, macrophages, monocytes, and natural killer T (NKT) cells. The role of these cells in different immune responses has led to the design of targeted immunotherapies aimed at controlling diseases, such as cancer, autoimmunity, and allergy, as well as potentiating immune responses against pathogens. In this special issue, original studies and review articles are encompassed together to highlight recent discoveries on immunotherapy involving innate immune cells to combat cancer and pathogens.

In the area of cancer immunotherapy, R. Yi et al. analyze how innate immune cell-based immunotherapy combined with classical therapies can improve the outcome of castration-resistant prostate cancer. They perform a meta-analysis aimed at studying the efficacy of the immunotherapeutic vaccine sipuleucel-T combined with regular treatments directed to the androgen receptor (AR). Sipuleucel-T is a cellular immunotherapy which consists in autologous peripheral blood mononuclear cells (PBMCs) incubated with recombinant specific prostatic antigens fused with granulocyte-macrophage colony-stimulating factor. They described that this immunotherapy greatly improved the efficacy of the traditional therapy. Also aiming at cancer, P. G. Lokhov and E. E. Balashova investigate the efficacy of targeting tumor vasculature antigens and their efficacy against cancer growth. They describe the novel composition called SANTAVAC (Set of All Natural Target Antigens for Vaccination Against Cancer) and their use against tumoral microvasculature. Finally, I. Knippertz et al. used a combined promoter system of adenoviral vectors in order to induce maturation changes in DCs. By targeting the active expression of heat shock factor (mHSF), they induced maturation of human DCs, with a concomitant increase in the expression of proinflammatory cytokines and costimulatory molecules. This approach, combined with specific antigens, could be useful not only in cancer immunotherapy but also in improving immunity against pathogens.

In the area of pathogen immunotherapy, X. Yu et al. constructed a bispecific antibody with the ability to interact with HIV and HIV-infected cells in one direction and with CD89 in the other. This led to the targeting of neutrophils and polymorphic mononuclear cells (PMNs) and resulted in optimal anti-HIV activity. Although this strategy has been described before, in the current work, the authors solved structural limitations of the previous bispecific antibodies, describing two novel structural-modified antibodies that show excellent activity at inhibiting HIV infection and mobilizing innate immune cells. Finally, S. Schülke et al. investigate the adjuvant potential of monophosphoryl lipid A (MPLA) when chemically coupled with protein antigens, using ovalbumin (OVA) as an example. They described that the coupled protein-adjuvant composition is not suitable for allergy treatment, but likely appropriate for other diseases, such as pathogen infections.

Taken together these articles represent novel advances in the field of innate cell-based immunotherapy for cancer and pathogen immunity and may be considered for future clinical studies. Altogether, we are certain that we will see an important increase in the numbers of studies focusing on innate immune cells for immunotherapy in the near future, as these approaches are showing promising results against important diseases.

